# Understanding the lineshape of surface-enhanced infrared absorption spectra

**DOI:** 10.1093/nsr/nwaa240

**Published:** 2020-09-23

**Authors:** Shunping Zhang, Hongxing Xu

**Affiliations:** School of Physics and Technology, Center for Nanoscience and Nanotechnology, and Key Laboratory of Artificial Micro- and Nano-structures of Ministry of Education, Wuhan University, China; School of Physics and Technology, Center for Nanoscience and Nanotechnology, and Key Laboratory of Artificial Micro- and Nano-structures of Ministry of Education, Wuhan University, China; The Institute for Advanced Studies, Wuhan University, China

Infrared absorption spectroscopy offers fingerprint identification of molecular analytes through their vibrational or rotational degrees of freedom. However, the absorption cross-section of a single molecule is very small because the size of the molecule is far smaller than the wavelength of light, typically ranging from 2.5 to 25 μm. Therefore, infrared absorption experiments always require a large number of target molecules within the detection volume. Surface-enhanced infrared absorption (SEIRA) spectroscopy is a technique that typically uses metallic nanostructures to amplify absorption of the molecule, enabling detection of the analytes down to a trace amount [1]. In most cases, this works via giant field enhancement in the vicinity of the metal surfaces, provided by the localized surface plasmons [2]. However, the lineshape of SEIRA usually becomes complicated as the coupling of molecular vibrations and the plasmon gets stronger, which hampers accurate readout of the vibrational frequencies and spectral intensities for chemical identification or quantitative analysis.

**Figure 1.**
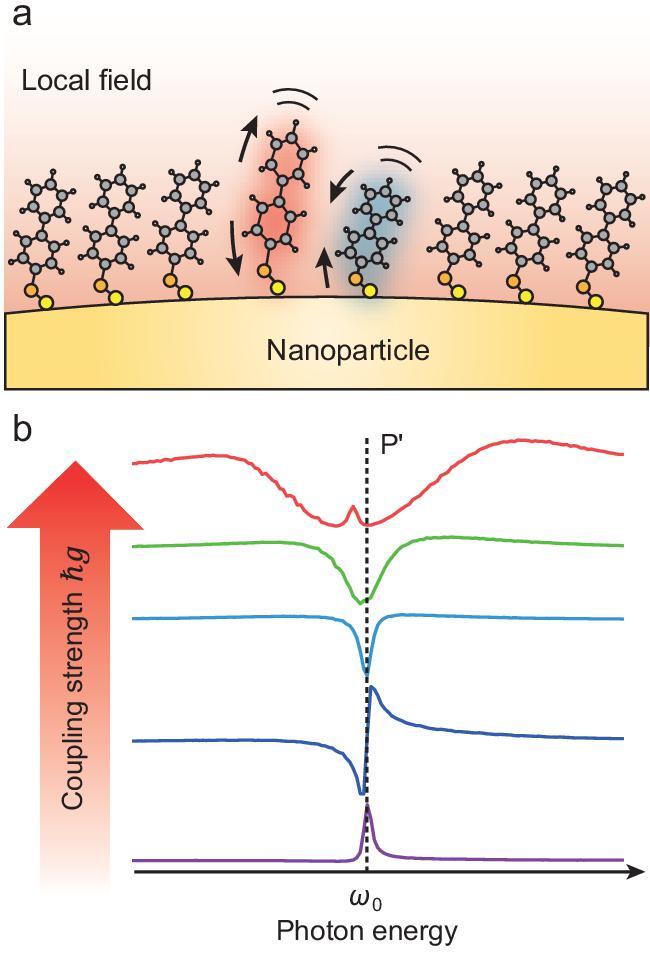
(a) Schematic plasmon-mediated intermolecular coupling between different molecules. (b) Lineshape evolution as the coupling strength increases [4].

Asymmetric lineshapes, usually Fano profiles, appear when the rate of energy exchange between the vibration and plasmon gets closer to the rates at which they dissipate (intermediate coupling regime). When the coupling strength is further increased to the strong coupling regime, the two peaks repulse each other, featuring an avoided-crossing behavior that is universal in interacting entities. The above transition from weak to strong coupling in SEIRA can be fully understood via the classical two coupled oscillators model [3], if there is only one molecule involved. As the number of molecules increases, the degree of freedom involved in the coupling is increased, and the description of the system by many oscillators becomes complicated. Jun Yi *et al.* from Xiamen University reproduced weak, intermediate, and strong coupling of the SEIRA system using a prototype of core-shell nanostructure [4]. They found that an additional peak arises when coupling between the plasmon and the N-molecule is sufficiently strong, and attributed this to plasmon-mediated intermolecular coupling (Fig. 1), an internal interaction that could not be reproduced by the two coupled oscillators model.

In quantum optics, the interaction of light with N atoms is usually modeled by the interaction of a photon with a giant atom resulting from coherent coupling of the N atoms. This assumption covers up all the internal interaction processes and always results in lineshapes with two peaks at most. However, if the number of molecules is infinite, the coupling system becomes simple again as it now behaves as a classical system. The molecular assembly can then be described by a classical continuous medium with Lorentz permittivity [5], which allows modeling of multipole interactions within the hybrid system. This is the physics behind the electromagnetic modelings used by the group from Xiamen. This work strengthens our understanding of molecular density-tuned interactions between molecular vibrations and plasmons, and will trigger further experimental and theoretical studies on coherent coupling between electronic or vibrational excitations from finite molecules and plasmonic excitations.

***Conflict of interest statement.*** None declared.
